# Low-Temperature Direct Oxidation of Propane to Propylene Oxide Using Supported Subnanometer Cu Clusters

**DOI:** 10.1021/acscatal.4c07577

**Published:** 2025-03-24

**Authors:** Avik Halder, Robert E. Warburton, Geng Sun, Lei Cheng, Rajeev S. Assary, Soenke Seifert, Micaela Homer, Jeffrey Greeley, Anastassia N. Alexandrova, Philippe Sautet, Larry A. Curtiss, Stefan Vajda

**Affiliations:** † Materials Science Division, Argonne National Laboratory, Lemont, Illinois 60439, United States; ‡ Davidson School of Chemical Engineering, Purdue University, Lafayette, Indiana 47907, United States; § Chemical and Biomolecular Engineering Department, University of California, Los Angeles, California, 90095, United States; ∥ Advanced Photon Source, Argonne National Laboratory, Lemont, Illinois 60439, United States; ⊥ Chemistry and Biochemistry Department, University of California, Los Angeles, California 90095, United States; # Department of Nanocatalysis, J. Heyrovský Institute of Physical Chemistry, Czech Academy of Sciences, 182 23 Prague 8, Czech Republic

**Keywords:** oxidative dehydrogenation, epoxidation, density functional calculations, propane, propylene, propylene oxide, subnanometer clusters, copper

## Abstract

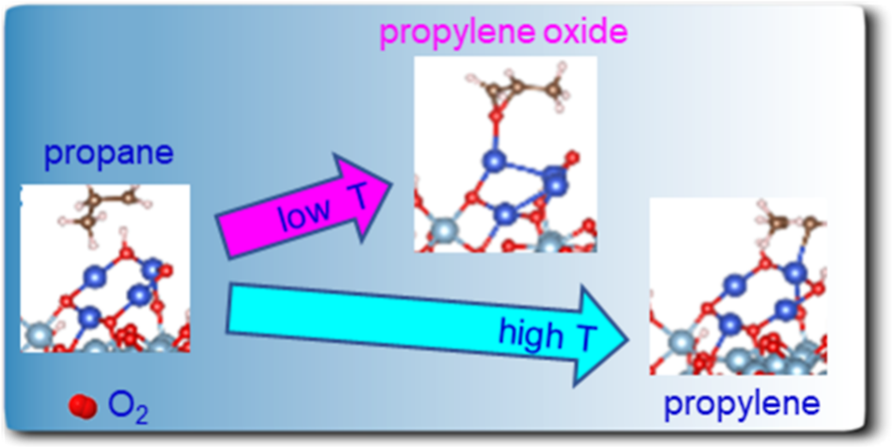

Propylene oxide, a key commodity of the chemical industry for a wide range of consumer products, is synthesized through sequential propane dehydrogenation and epoxidation reactions. However, the lack of a direct catalytic route from propane to propylene oxide reduces efficiency and represents a major challenge for catalysis science. Herein, we report the discovery of a highly active and selective catalyst, made of alumina-supported subnanometer copper clusters, which can directly convert propane to propylene oxide at temperatures as low as 150 °C. Moreover, at higher temperatures, on the same catalysts, the selectivity is switched to propylene. Accompanying theoretical calculations indicate that partially oxidized and/or hydroxylated clusters have low activation energies for both propane dehydrogenation and propylene epoxidation pathways, enabling direct conversion with very high selectivity for propylene oxide. The discovery of a low-temperature catalyst that can convert propane directly to propylene oxide provides an important opportunity for the development of energy-efficient and economic catalysts for this industrially critical process. Similarly, when operating at higher temperatures, these catalysts are posed as potent oxidative dehydrogenation catalysts.

## Introduction

Propylene oxide (PO) is a key commodity chemical, typically produced from the epoxidation of a propylene feedstock, and is used to produce a wide range of consumer products such as rigid foams, moldings, adhesives, and coatings.^1,2^ Industrial techniques for indirect synthesis of PO, based on chlorohydrin, hydroperoxide, cumene, and styrene monomers, produce abundant side products.^3−5^ More direct routes to produce PO include propylene epoxidation.^6−9^ In the latter case, propane is first dehydrogenated to propylene, and then propylene is selectively oxidized to PO. There have been a large number of works published over the years, and both processes, propane to propylene as well as propylene epoxidation, remain among the most studied reactions due to the steadily increasing demand for propylene and PO. A wide spectrum of metal- and metal oxide-based catalysts,^10,11^ photocatalyst,^12^ electroassisted/electrocatalysts,^13^ and plasmon-enhanced catalysts^14^ have been investigated for propane dehydrogenation, including the use of CO_2_ as the oxidant,^15^ for the second step of propylene epoxidation,^8,9,16−19^ including the limitations of the discussed processes in terms of used material, operating temperature, or stability,^20,21^ just to pick a handful of recent reviews and papers for the discussed processes. For both reactions, the route of direct oxidation remains the most preferred one, together with nonprecious metal- or metal oxide-based catalyst, from an energetic and cost perspective.

Reports of catalysts capable of producing PO directly from propane are extremely scarce. In the oxidation of propane at elevated temperatures between 390 and 500 °C performed on boron nitride and silicon dioxide, in addition to the main product propylene, PO was detected as well, with up to about 10% selectivity.^22^ We decided to investigate subnanometer clusters because they have previously been found to be catalytically highly active for propane oxidative dehydrogenation and propylene epoxidation separately.^7,23,24^ We chose to study Cu clusters because they are highly active for the two individual catalytic reactions.^25−27^ Herein, we report on our finding that alumina-supported subnanometer Cu clusters can produce PO directly from propane. Moreover, these catalysts turn over propane to PO at a high rate and with exceptional selectivity at temperatures between 150 and 300 °C and suppress combustion of the feed.

## Materials and Experimental Methods

As support material, a ∼3 ML thin film of alumina is used, prepared by atomic layer deposition on the native oxide on the top of the n-type (P-doped) Si wafer. The catalysts are then prepared by soft landing of ligand-free atomically precise 4-, 12-, or 20-atom copper clusters (Cu_4_, Cu_12_, or Cu_20_). A cluster beam is produced by magnetron sputtering in a vacuum apparatus,^28^ and the emerging molecular beam containing positively charged Cu clusters of a wide distribution of sizes passes through a quadrupole mass filter on which clusters of desired atomicity are mass selected. The clusters of single size are then landed on the support with a controlled kinetic energy so that the impact energy during landing is lower than 1 eV per atom to ensure that the clusters stay intact and do not undergo fragmentation or pinning on the substrate. There are two cluster spots of 8 mm diameter each, deposited on the support of 20 × 22 mm, at a metal coverage corresponding to 10% of the atomic monolayer equivalent. The coverage, i.e., the amount of deposited metal, is determined via integration of charge over the deposition time of the current of charged clusters arriving on the surface. In the knowledge of the accumulated charge and of a single charge by a cluster of given atomicity, the exact number of deposited clusters and atoms is determined. Reactivity data are collected on a mass spectrometer for both the cluster-containing samples as well as the blank, i.e., cluster-free support under identical conditions. The reactivity data collected for the blank support are then subtracted from that of the cluster sample, yielding a difference that corresponds to the pure reactivity by clusters only, i.e., background and support-free. Using the concentrations of the given product after calibration, together with the known number of deposited Cu atoms, the rate of production of a given molecule per deposited total Cu atom and per unit time is finally calculated. The reaction products are identified from mass spectra based on characteristic fragmentation patterns of the product molecules and the evolution of the intensity of the selected fragment peak (i.e., *m*/*z*) with temperature followed during the temperature ramp. The concentration of the individual products is determined from the calibration of the mass spectrometer to the individual products. To quantify the reaction products, the sensitivity factors of the mass spectrometer for individual molecules were calculated by using calibrated gas mixtures with the given molecule in helium carrier gas (certified analytical grade mixed gases, Airgas Inc.). The uncertainty is estimated to be ∼2% of the ion current. Taking into consideration an estimated 10% uncertainty in the determination of the number of deposited atoms, the error in the determination of the rates is estimated to be about 10%,^29^ with data processing, yielding the rates, described in details in ref (30).

The catalytic reaction is investigated under *operando* conditions in a custom reactor,^31^ in which the reactivity data are collected on a mass spectrometer as well as X-ray absorption and small-angle X-ray scattering data in a surface-sensitive grazing-incidence geometry (GIXAS and GISAXS), the latter to gain information about the nature of Cu and the sintering-resistance of the clusters under working conditions, respectively, at a pressure of 1.1 atm, with a reactant gas mixture consisting of 2% of propane and 2% of oxygen seeded in helium, fed into the reaction cell of 30 cm^3^ volume at a flow rate of 18 sccm. In the cell, the sample is placed on the top of a boron-nitride heater; the temperatures applied are 25, 50, 150, 300, 400, 450, 500, and 550 °C, with a slow heating 5 °C min ^–1^ between the individual temperature steps to ensure thermal stabilization of the sample, and with 30 min dwell time at each temperature step to collect reactivity data, X-ray absorption, and X-ray scattering data.^31^

## Computational Methods

Periodic, plane-wave, spin polarized density functional theory (DFT) calculations were performed using the Vienna Ab initio Simulation Package (VASP).^32,33^ The generalized gradient approximation of Perdew and Wang (GGA-PW91) is used as the exchange and correlation functional.^34^ Core states are represented using pseudopotentials based on the projector augmented wave method^35,36^ and the valence states are expanded in a plane-wave basis set with a kinetic energy cutoff of 400 eV. Our calculations use the model of Cu_4_O_4_ supported on amorphous hydroxylated alumina reported previously,^37^ using a vacuum space of 20 Å with electrostatic dipole corrections apply at the center of the vacuum layer. We use 2 × 2 × 1 Γ-centered *k*-point mesh, and the total electronic energies are converged to a tolerance of 10^–4^ eV per unit cell. The geometries are subject to a force criterion of 50 meV Å^–1^. Transition states are identified using the climbing image nudged elastic band method to determine the minimum energy path (MEP).^38,39^ We also use the image-dependent pair potential method to enable efficient, chemically reasonable, initial guess structures for intermediate images along the MEP.^40^ The DIMER method^41^ is used to refine the transition state energies, and we confirm a saddle point with the calculation of a single imaginary frequency corresponding to the degree of freedom along the MEP reaction coordinate.

We applied the Campbell–Sellers relationship^42^ to calculate entropic corrections associated with C_3_H_6_ desorption and assumed minimal entropic contributions from high-frequency hydroxyl vibrations and oxygen vacancy creation, such that the entropy gain from dehydration is solely described by the entropy of gas-phase H_2_O.

Calculations for the gas phase clusters (Figure S6) are performed using Gaussian 09 software [9].

Global optimizations are carried out for exploring the optimal structures of supported hydroxylated Cu oxide clusters on a partially hydroxylated amorphous alumina support. The support structure is obtained by force-field-based molecular dynamic simulation, which was previously reported in the literature. In order to save the computational cost, the thickness of the slab was truncated and saturated by (pseudo) hydrogen atoms whose core charges are 0.25, 0.5, 0.75, 1.0, and 1.25, respectively, ensuring the total net charge of the slab is zero. The coordinates of the slab are shown in Supporting Information. In total, 9 different compositions are considered in the global optimization, and the composition can be notated by Cu_4_O_*x*_(H_2_O)_*y*_. The number of oxygen (*x*) is 2, 3 and 4, respectively, and the number of additional water molecules (*y*) is 0, 1, and 2, respectively. The global optimizations were conducted by an in-house package using the Basin Hopping algorithm, and the VASP was used as the workhorse for conducting the DFT computations. Spin-polarized simulations are used throughout this study. A Hubbard (*U* = 7.0) term is exploited for the d orbital of the Cu atoms to mitigate the self-interaction error in the PBE functional, according to our previous study of a similar model^43^ using the hybrid functional HSE06 as a reference.

The stability of the Cu_4_O_*x*_(H_2_O)_*y*_ clusters is analyzed by the ab initio thermodynamics method. The formation free energy of the cluster is evaluated as
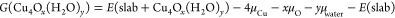
1in eq 1, the free energies of the condensed phase (i.e., the bare slab and the cluster-deposited slab) are estimated by their electronic energies. Since we are only interested in the relative stabilities of clusters of different stoichiometries, the constant terms (μ_Cu_ and *E*(slab)) are ignored in the subsequent analysis.

We take the chemical potential of oxygen at the standard condition (1 bar and 25 Celsius) as μ^θ^(O) = −5.31 eV.^43^ We defined the difference between μ^θ^(O) and electronic energy of O_2_ as a thermodynamic correction, which should be a constant among different DFT codes:
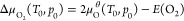
2where *T*_0_ = 25 °C and *p*_0_ = 1 bar. Therefore,
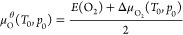
3the difference between the chemical potential of oxygen Δμ(*T*, *p*) at any *T*, *p* condition, and the standard condition (25 °C, 1 bar) is evaluated by the ideal gas model, implemented in the ASE package.

Finally, the chemical potential of oxygen μ(*T*, *p*) is calculated by
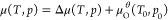
4

For water molecules, since DFT works pretty well for evaluating the binding energies, we calculate the chemical potential of water simply by ideal gas model. The difference Δμ_H_2_O_ is the difference between the evaluated free energy and electronic energy:
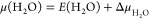
5

In exploring the reaction pathways, we corrected the van der Waals (vdW) interaction by the DFT-D3 method. This is justified by the fact that the vdW interaction is critical only when evaluating the weak interaction between the catalyst and the hydrocarbons.

## Results and Discussion

**Figure 1:**
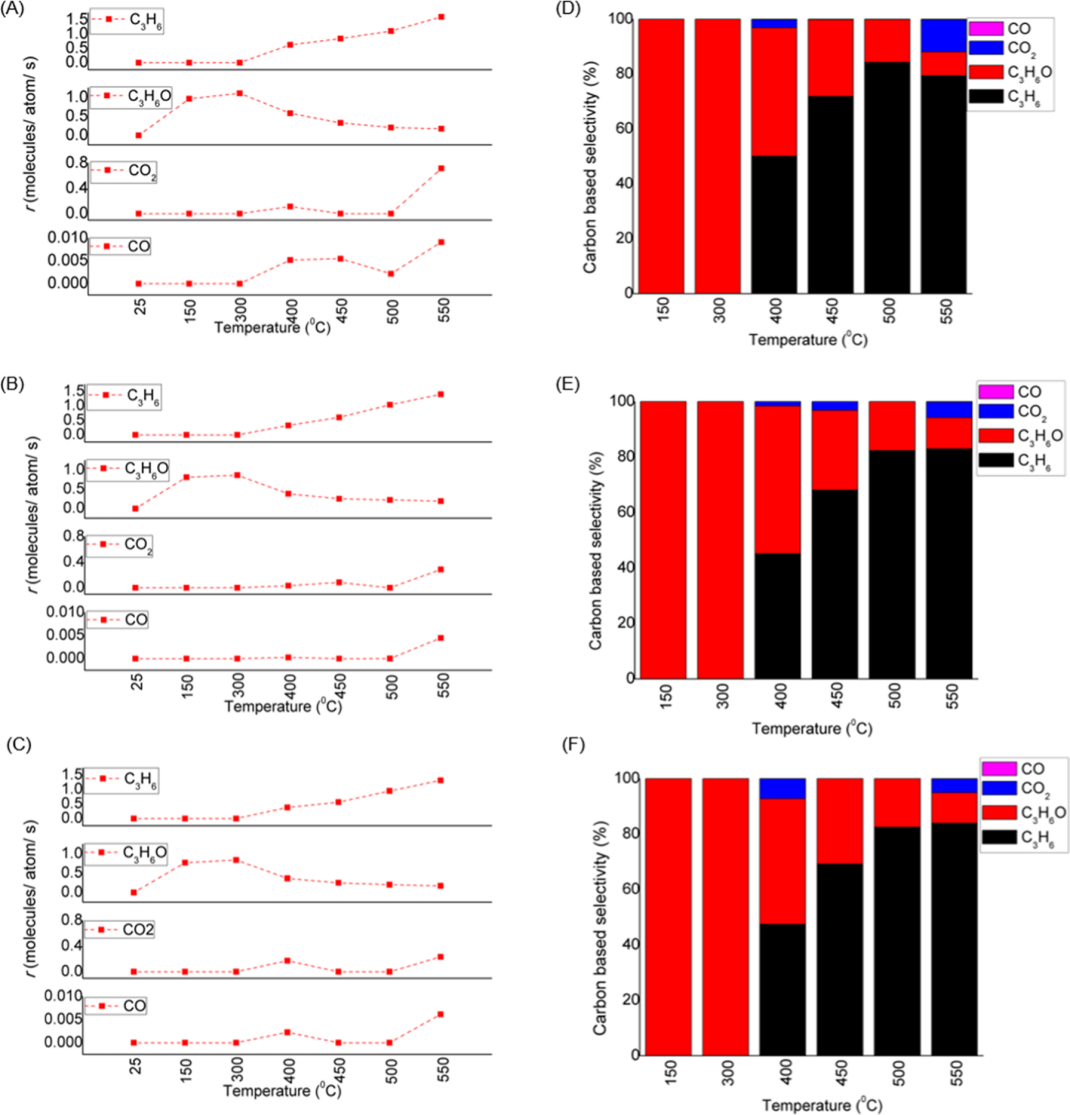
Rates of formation of propylene, propylene oxide, and byproducts CO, and CO_2_ for alumina-supported Cu_4_ (A), Cu_12_ (B), and Cu_20_ (C) clusters. Carbon-based selectivity for the reaction products are plotted for Cu_4_ (D), Cu_12_ (E), and Cu_20_ (F) cluster samples.

From the mass spectrometer data collected for Cu_4_, we find that the PO signal (*m*/*z* = 58) appears at 150 °C and remains high until reaching 300 °C (see Figure 1 for the evolution of the products, plotted as the reaction rate (*r*), defined as the number of product molecules formed per Cu atom on the catalyst per second). At temperatures above 300 °C, the PO signal drops, and a rise in propylene (*m*/*z* = 29), CO (*m*/*z* = 28), and CO_2_ (*m*/*z* = 44) is observed.

The reaction rates (*r*) for the three investigated Cu cluster sizes with 4, 12, and 20 atoms are presented in Figure 1a–c, respectively. For Cu_4_, the PO production reaches a rate of 1.2 molecules/second/atom at 300 °C (see Figure 1a). The rate of formation of PO matches is significantly higher than that obtained by currently used techniques where propylene or a mixture of propane and propylene is used as the feed.^5,8,44,45^ These comparisons further amplify the high efficacy of the Cu_4_ clusters for converting propane directly to PO. Notable is also the high selectivity (∼100%) toward PO at temperatures between 150 and 300 °C. The rates obtained for the larger Cu clusters, Cu_12_, and Cu_20_, are comparable to those observed for Cu_4_ clusters (see Figure 1b,c). This result suggests that in the studied size range the performance of these catalysts is comparable, the cluster size is not critical, thus, potentially opening possibilities of similar Cu cluster-based catalysts fabricated with a size distribution by wet chemistry or other more traditional synthesis methods^46^ suitable for the large scale production of the catalyst. The observed dip in the rate of CO_2_ formation at around 500 °C coincides with the increasing rate of propylene formation for all three investigated cluster sizes. With the rate of CO_2_ formation becoming at 550 °C the highest for the 4-atom cluster and dropping with cluster size, we hypothesize that this trend possibly reflects a cluster size/structure effect on selectivity.

In order to explore the effect of the support material on catalytic performance, we deposited identical Cu_4_ clusters on carbon-based ultrananocrystalline diamond (UNCD) supports as well.^47^ On this carbon-based support the reaction rate between 150 and 300 °C dropped by about an order of magnitude compared to that on alumina support (see Figure S1). While the oxidation state of Cu in the UNCD-supported clusters was comparable to that on Al_2_O_3_ (see Figure S2) as discussed below, this significant difference in performance underlines the important role of cluster-support interactions and the central role of the interface between the cluster and oxide support in tuning the catalytic performance of ultrasmall clusters. For the propylene to PO reaction with Ag clusters, we previously found a similar support effect and attributed it to the larger DFT-calculated barrier for O_2_ dissociation at the UNCD/Ag cluster interface compared to at the Al_2_O_3_/Ag cluster interface.^48^

**Figure 2:**
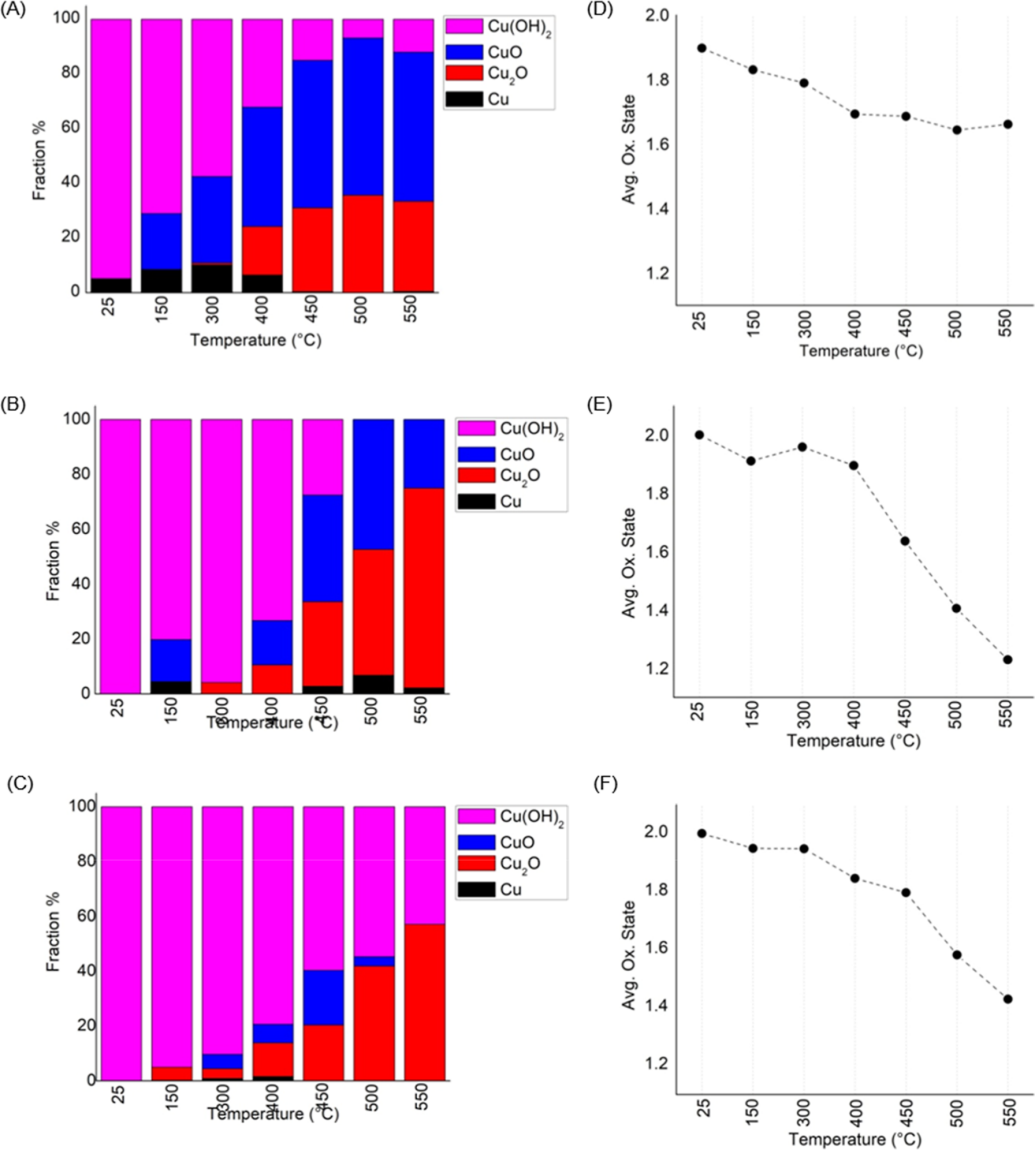
Results from LCF analysis of Cu K-edge XANES spectra for alumina-supported Cu_4_, Cu_12_, and Cu_20_ clusters. (A–C) Cu K-edge of the supported Cu clusters used to estimate the oxidation state of Cu (D–F): (A,D) Cu_4_, (B,E) Cu_12_, and (C,F) Cu_20_ cluster catalysts.

In order to probe the nature of the working alumina-supported Cu, we employed *operando*, grazing incidence X-ray absorption near edge spectroscopy (GIXANES) and grazing incidence small-angle X-ray scattering (GISAXS).^31^ The evolution of the composition and oxidation state of Cu during the applied temperature ramp (see Figure 2) was obtained by linear combination fitting (LCF) of the XANES spectra of the clusters (Figure S3), to the spectra of Cu bulk standards Cu, Cu_2_O, CuO, and Cu(OH)_2_ (Figure S4). The LCF analysis on the spectra of the Cu_4_ sample suggested a dominating Cu(OH)_2_ composition at the beginning of the temperature ramp, with Cu in an average oxidation state close to +2, as shown in Figure 2a,b. Above 100 °C, a steady change in the composition of Cu was observed, with a drop in the fraction of the Cu(OH)_2_ component, accompanied by a rise in CuO, as shown in Figure 2a. The average oxidation state of Cu_4_ exhibited a minor drop from +1.9 to +1.7 at 400 °C, and stayed unchanged thereafter with further increases in temperature. In the case of Cu_12_ and Cu_20_, Cu reduced from an initial average oxidation state of + ∼2 to +1.2 and +1.4, respectively, moreover with a significantly increased fraction of the hydroxide component in both clusters. We observed such a size-dependent trend in the reduction of Cu^49^ also during the conversion of CO_2_ to methanol on alumina-supported Cu clusters. For comparison with the alumina-supported tetramer, XANES spectra of Cu_4_ supported on nanocrystalline diamond collected under identical reaction conditions revealed a more reduced Cu with an oxidation state of + ∼1.6 without exhibiting a temperature dependency (see Figure S4), thus indicating weak, if any, cluster-support interactions and differing properties of the cluster-support interface. GISAXS indicated no signs of sintering of clusters during the reaction (see Figure S5).

In order to understand the molecular underpinnings of the high activity and temperature-dependent selectivity of the Cu_4_ clusters, we performed periodic, self-consistent density functional theory (DFT) calculations to elucidate the structures and compositions of Cu clusters in reaction conditions and evaluate mechanisms of direct propane conversion through oxidative dehydrogenation to propylene and subsequent propylene epoxidation. Based on the GIXANES results showing that Cu is divalent during the reaction, we first model these reactions with a Cu_4_O_4_ cluster on a hydroxylated amorphous alumina support. The alumina model was developed in previous work.^50^

**Figure 3:**
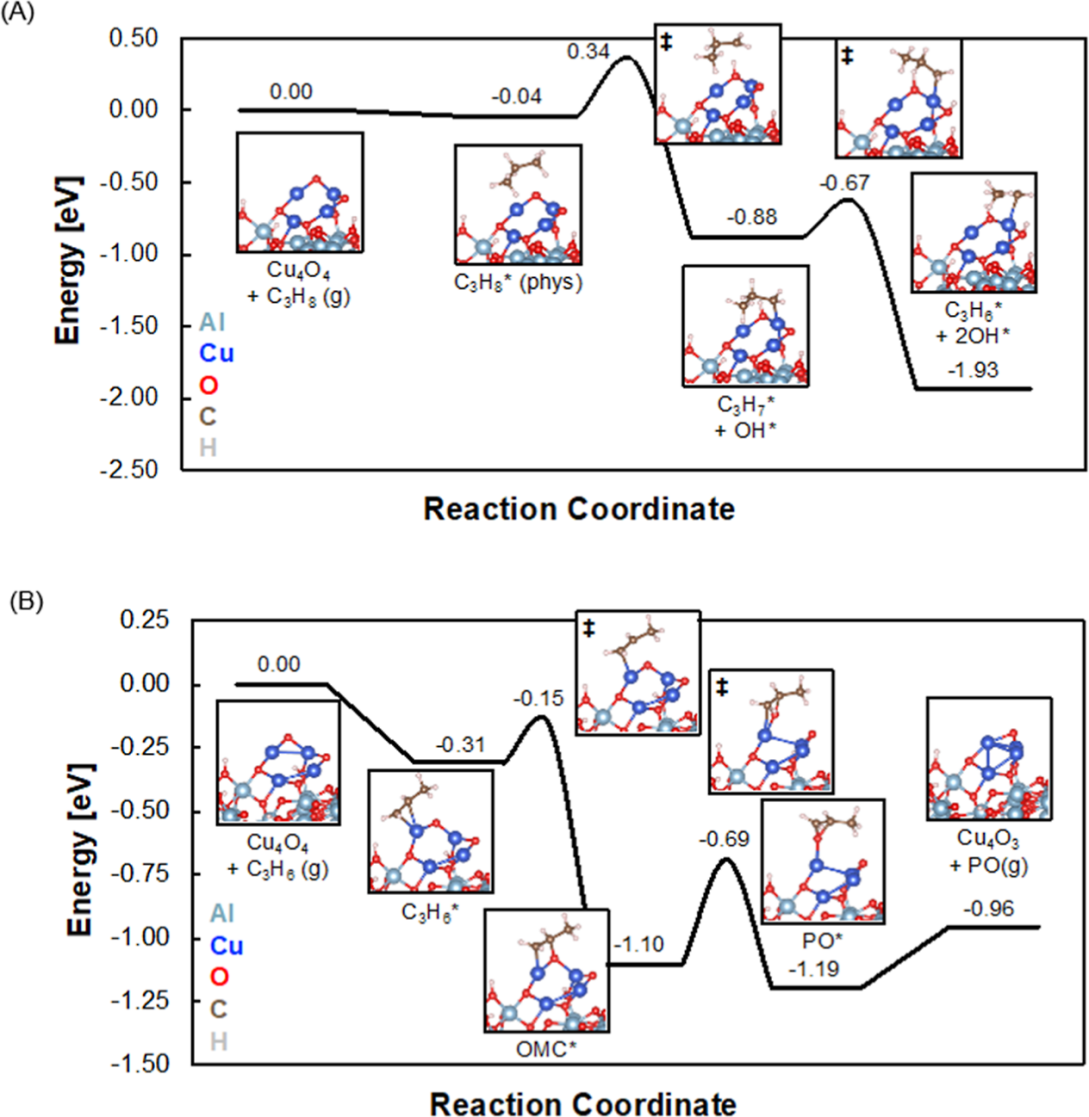
(A) Energies of intermediates and transition states for propane dehydrogenation to propylene. (B) Energies of intermediates and transition states for propylene epoxidation to PO.

Oxidative dehydrogenation occurs through two sequential hydrogen abstraction steps, forming propylene from propane by way of a propyl (C_3_H_7_) intermediate. Initial calculations were done on unsupported Cu_4_O_4_ clusters (see Figure S6), where we calculated barriers of 0.3 and 0.4 eV for the two hydrogen abstraction steps. Periodic DFT calculations on supported clusters gave results similar to the gas phase results. Figure 3a shows the intermediate structures on the alumina-supported Cu_4_O_4_ cluster as well as their calculated ground state energies and kinetic barriers.

C_3_H_8_ weakly physisorbs on the Cu_4_O_4_ cluster. The first hydrogen abstraction step then proceeds at a 0.38 eV intrinsic barrier. This results in the coadsorption of the 2-propyl (C_3_H_7_) intermediate with a hydroxyl group (OH) formed from hydrogen abstraction by dissociated oxygen on Cu. The second hydrogen abstraction, to form coadsorbed propene and a second OH group, has a barrier of only 0.21 eV relative to the adsorbed 2-propyl (C_3_H_7_) intermediate, making each of these dehydrogenation steps quite accessible at 100 °C. We find that the combined desorption of C_3_H_6_, accompanied by H diffusion to an OH group to desorb water to form a Cu_4_O_3_ cluster, is uphill in free energy by only about 0.09 eV. The ODH catalytic cycle may be closed through a combination of further C_3_H_8_ dehydrogenation or hydroxyl transfer to the reduced cluster, in addition to the dissociation of O_2_ to regenerate the supported Cu_4_O_4_ cluster. We find a low calculated barrier for O_2_ dissociation on the amorphous alumina-supported Cu oxide clusters needed for the subsequent epoxidation reaction (see Figure S7).

Figure 3b shows the C_3_H_6_ epoxidation pathway on a hydroxylated amorphous alumina-supported Cu_4_O_4_ catalyst. Considering the mechanistic analysis on Al_2_O_3_-supported Ag_3_ clusters in our previous work,^7^ PO is likely to be formed through an oxymetallocycle (OMC) intermediate as suggested for Cu,^51^ wherein an oxygen from the cluster reacts with the carbon double bond of C_3_H_6_.

The C_3_H_6_ has a binding energy of 0.31 eV to the Cu_4_O_4_ cluster, with the sp^2^ carbons bridging a single Cu atom, as shown in Figure 3. Forming the OMC intermediate requires diffusion of the C_3_H_6_* such that one of the carbon atoms remains bound to Cu, and the other forms a bond to an oxygen atom on the cluster. We find that the OMC formation proceeds through an accessibly low kinetic barrier of 0.16 eV, while the thermodynamics of this step are downhill by 0.79 eV. The subsequent kinetic barrier to form adsorbed PO is 0.41 eV, breaking both Cu–C and Cu–O bonds, and forming a Cu–Cu bond due to the presence of an oxygen vacancy. The PO desorption and cluster reduction to a Cu_4_O_3_ stoichiometry are modestly endothermic by 0.23 eV, and a calculated entropy gain for PO desorption of 0.44 eV at 100 °C suggests that PO desorption is facile.

**Figure 4:**
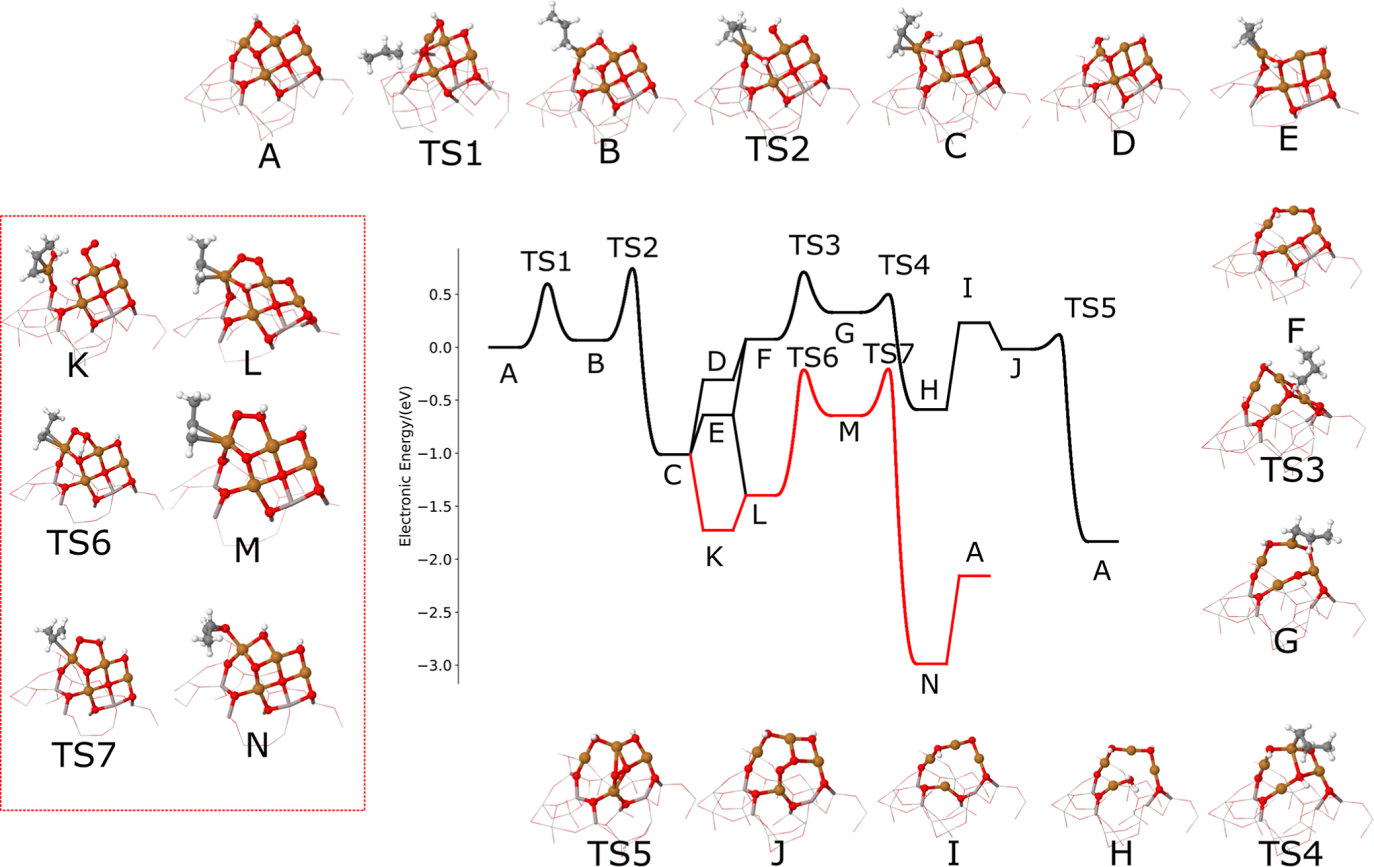
Oxidative dehydrogenation and epoxidation pathways on the hydroxylated catalyst whose formula is Cu_4_O_3_(OH)_2_ (structure A) supported on amorphous alumina. The pathways in red indicates the mechanism to propylene oxide, and pathways in black produce propylene.

Given some uncertainty in determining the stoichiometry of the clusters from *operando* XANES fitted to bulk standards,^52^ and also given that the kinetics of the PO reaction could lead to modest changes in steady-state oxidation states of the catalyst, we next explored the effect of a range of compositions of supported Cu_4_(O)_*x*_(OH)_*y*_ clusters. We analyzed the thermodynamic stability of a range of such clusters by constructing a phase diagram at realistic temperatures and O_2_ and water pressures using Basin Hopping global optimizations. This diagram shows that the clusters are partially hydroxylated up to a 300 °C temperature (P(O_2_) = 0.1–1 bar and P(H_2_O)/P(O_2_) 0.01–0.1) (Figure S8). The proposed catalyst model is labeled as A in Figure 4 (the formula is Cu_4_O_3_(OH)_2_, corresponding to the formal oxidation state of Cu of +2, though both the Cu and the O atoms interact with the support, complicating the oxidation state assignment). At higher temperatures, the clusters are confirmed to contain no hydroxyls, as shown in Figure 3. Although kinetic steady states may lead to small changes in cluster structure compared to the thermodynamic predictions, the broad range of considered structures will be representative of all plausible surface states.

A detailed reaction pathway on catalyst A was additionally constructed, including propane oxidative dehydrogenation to propylene, followed by competitive propylene desorption or epoxidation (Figure 4). The chemical formula of the proposed partially hydroxylated structure of the active catalyst can be expressed as Cu_4_O_3_(OH)_2_ (excluding the alumina support). The propane is activated by a three-coordinated oxygen from the active catalyst, and the reaction barrier is moderate and finally produces a propyl on the Cu and another hydroxyl on the catalyst. The second C–H bond breaking takes place from the β carbon, and a surface hydroxyl is further hydrogenated to a water molecule. The second C–H bond breaking is strongly exothermic. The water and formed propene can desorb stepwise and a reduced Cu_4_O_2_(OH)_2_ cluster results. The epoxidation mechanisms are altered from Figure 3 (the red part of the profile on Figure 4): starting from the adsorbed propylene intermediate, O_2_ first adsorbs, rearranges from η_1_ to η_2_, O_2_–η_2_ is then protonated and this O–OH group can react with propylene to make the epoxide, leaving an OH group on the cluster and hence restoring the initial Cu_4_O_3_(OH)_2_ catalyst structure. In the competing pathway where propylene instead desorbs, a second propane oxidative dehydrogenation reaction takes place to yield propylene and a Cu_4_O(OH)_2_ cluster, which then reacts with gas phase O_2_ to restore Cu_4_O_3_(OH)_2_. The surface hydroxylation affects the epoxidation selectivity in twofold: first, the cluster is slightly passivated by hydroxylation, leading to higher barriers for C–H cleavage (as seen in the higher barriers for first and second C–H cleavage); second, the protonation of adsorbed O_2_ forming an OOH weakens the O–O bond, rendering epoxidation faster and increasing the selectivity toward the epoxide. Therefore, the hydroxylated model is active both for propane ODH and for epoxidation, and a switch from the hydroxylated model to the dry model is expected at high temperature. In both hydroxylated and nonhydroxylated (Figure 3) cases, the cluster will exhibit high reactivity for PO formation.

Although our experimental efforts suggest that at low temperatures, the only major product formed on Cu_4_ is PO, we nevertheless also consider the formation of acrolein (C_3_H_4_O) in our computational analysis. In contrast to the epoxidation pathway, the acrolein formation pathway on a Cu_4_O_4_ cluster requires two hydrogen abstraction steps (see Figure S9). Following the adsorption of C_3_H_6_, the first hydrogen abstraction barrier is 0.40 eV to form a coadsorbed OH group and C_3_H_5_. The C_3_H_5_* then forms an oxygen transfer intermediate represented by C_3_H_5_O* in Figure S9 with a low kinetic barrier of 0.19 eV. The last step to form acrolein, C_3_H_4_O*, involves a second hydrogen abstraction, requiring a barrier of 1.16 eV. This barrier is not only quite high, but the formation of acrolein may also be inhibited by shifts in reaction equilibria from H_2_O formation in the ODH pathway, i.e., Le Chatelier’s principle.

## Conclusions

In summary, we have shown that supported subnanometer Cu clusters are active for the direct conversion of propane to PO at low temperatures with high turnover rates and selectivity while suppressing the formation of the combustion products CO and CO_2_. Accompanying theoretical calculations provide a mechanistic understanding of the facile reaction pathways for propane dehydrogenation and propylene epoxidation mechanisms on dry or hydroxylated clusters, since the hydroxylated cluster is present at temperature below 300 °C from our theoretical phase diagram and detected via XANES.

The discovery that Cu clusters can act as low-temperature catalysts for the conversion of propane directly to PO provides an important opportunity for the development of new catalysts for this industrially critical process.

While the original aim of the study was to identify a catalyst for the direct production of PO from propane, these same catalysts convert propane to propylene with high efficiency and selectivity at higher temperatures, thus switching selectivity with temperature and moreover with the production of PO or propylene at high rates. The results from theoretical calculations underline the related mechanisms in PO vs propylene formation, based on the role of hydroxylated and dry catalytic moieties.

## Data Availability

Data are available from the corresponding authors upon request.

## Supporting Information Available

Rate of formation and carbon-based selectivity for reaction products over Cu_4_ clusters on UNCD support; GIXANES spectra of supported Cu clusters; XANES spectra of bulk Cu standards; XANES analysis for Cu4 clusters on UNCD; GISAXS spectra of supported Cu clusters; computed energy profile for the gas phase conversion of propane to propene by Cu_4_O_4_; O_2_ dissociation on hydroxylated amorphous alumina-supported Cu_4_O_2_; phase diagram of the hydroxylated Cu oxide cluster; and energies of intermediates and transition states for the side reaction of acrolein formation from propylene (PDF)
