# Supplementary material for: Low-Temperature Direct Oxidation of Propane to Propylene Oxide Using Supported Subnanometer Cu Clusters
Source: ACS Catal. 2025 Mar 24;15(7):5760–9. doi: 10.1021/acscatal.4c07577 (PMC11976697; doi:10.1021/acscatal.4c07577)
Supplement: Supplementary file 1 — cs4c07577_si_001.pdf [file cs4c07577_si_001.pdf]

Supporting Information

for

Low-temperature direct oxidation of propane to propylene oxide using supported subnanometer Cu clusters

Avik Halder [1,€], Robert E. Warburton,[2,€,λ] Geng Sun[3€], Lei Cheng,[1] Rajeev S. Assary,[1] Soenke Seifert[4], Micaela Homer[1], Jeffrey Greeley[2], Anastassia N. Alexandrova[5*], Philippe Sautet[3,5]*, Larry A. Curtiss[1*], Stefan Vajda[1,6*]

[1] Materials Science Division, Argonne National Laboratory, Lemont, IL 60439, USA

[2] Davidson School of Chemical Engineering, Purdue University, Lafayette, IN 47907, USA

[3] Chemical and Biomolecular Engineering Department, University of California, Los Angeles, Los Angeles, CA 90095, USA

[4] Advanced Photon Source, Argonne National Laboratory, Lemont, IL 60439, USA

[5]Chemistry and Biochemistry Department, University of California, Los Angeles, Los Angeles, CA 90095, USA

[6] Department of Nanocatalysis, J. Heyrovský Institute of Physical Chemistry, Czech Academy of Sciences, 18223 Prague 8, Czech Republic

[€]Equally contributing authors

[λ]Current address: Department of Chemical and Biomolecular Engineering, Case Western Reserve University, Cleveland, OH 44106, USA

*Corresponding authors: curtiss@anl.gov (L.A.C.), ana@chem.ucla.edu (A.N.A.), sautet@ucla.edu (P.S.) and stefan.vajda@jh-inst.cas.cz (S.V.)

Table of Content:

Figure S1. Rate of formation and carbon based selectivity for reaction products propylene, propylene oxide and the byproducts CO, and CO_2 over Cu_4 clusters on UNCD support.

Figure S2. GIXANES spectra of supported Cu clusters of different sizes during oxidative dehydrogenation of propane collected at the Cu K-edge.

Figure S3. Cu K-edge XANES spectra of bulk Cu standards used for linear combination fit of the Cu K-edge spectra of Cu_n clusters.

Figure S4. XANES analysis for Cu_4 clusters on UNCD supports at Cu K-edge.

Figure S5. GISAXS spectra of supported Cu clusters during oxidative dehydrogenation of propane collected at 9.1 keV

Figure S6. Computed total energy profile for the gas phase conversion of propane to propene by a Cu_4O_4 catalyst in the gas phase.

Figure S7. O_2 dissociation on hydroxylated amorphous alumina-supported Cu_4O_2, chosen as a representative model for the oxidized clusters, to recover the Cu_4O_4 cluster stoichiometry.

Figure S8. Phase diagram of the hydroxylated copper oxide cluster.

Figure S9. Energies of intermediates and transition states for the side reaction of acrolein formation from propylene.

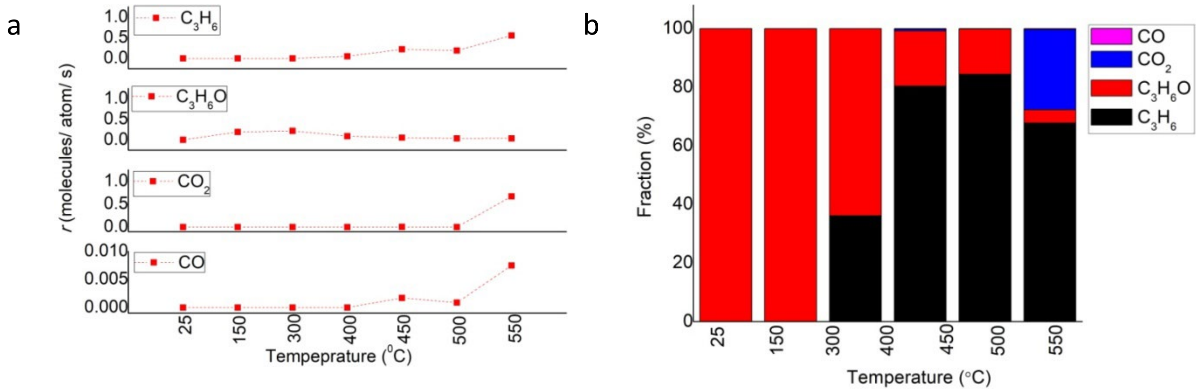

Figure. S1. Rate of formation (a) and carbon based selectivity (b) for reaction products propylene, propylene oxide and the byproducts CO, and CO_2during oxidative dehydrogenation of propane over Cu_4 clusters on UNCD support (2%propane and 2% oxygen in He under 1.1 atm).

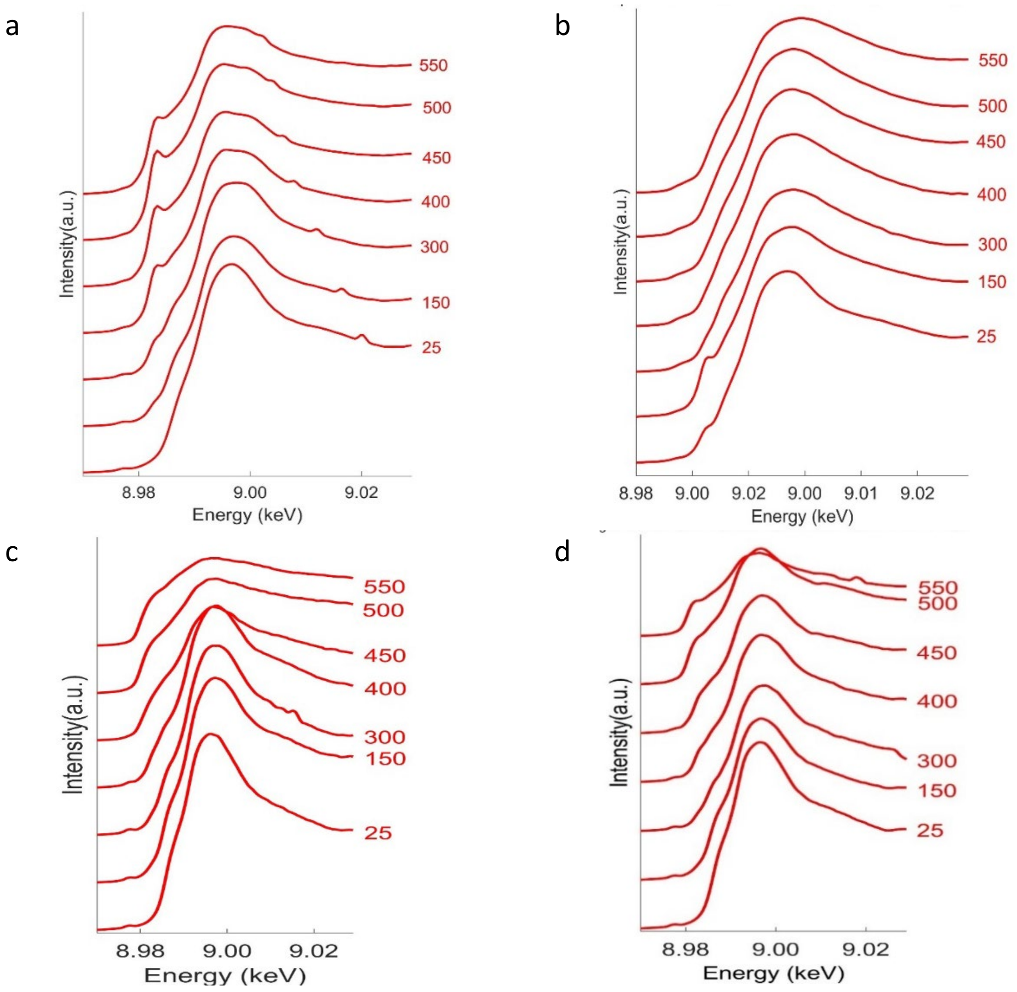

Figure S2. GIXANES spectra of supported Cu clusters of different sizes during oxidative dehydrogenation of propane collected at the Cu K-edge (2%propane and 2% oxygen in He under 1.1 atm). XANES spectra for (a) Cu_4 on alumina, (b) Cu_4 on UNCD, (c) Cu_{12} on alumina, and (d) Cu_{20} on alumina.

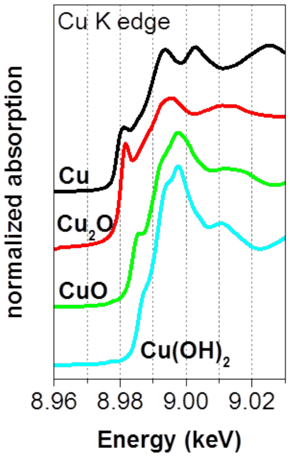

Figure S3. Cu K-edge XANES spectra of bulk Cu standards used for linear combination fit of the Cu K-edge spectra of Cu_n clusters

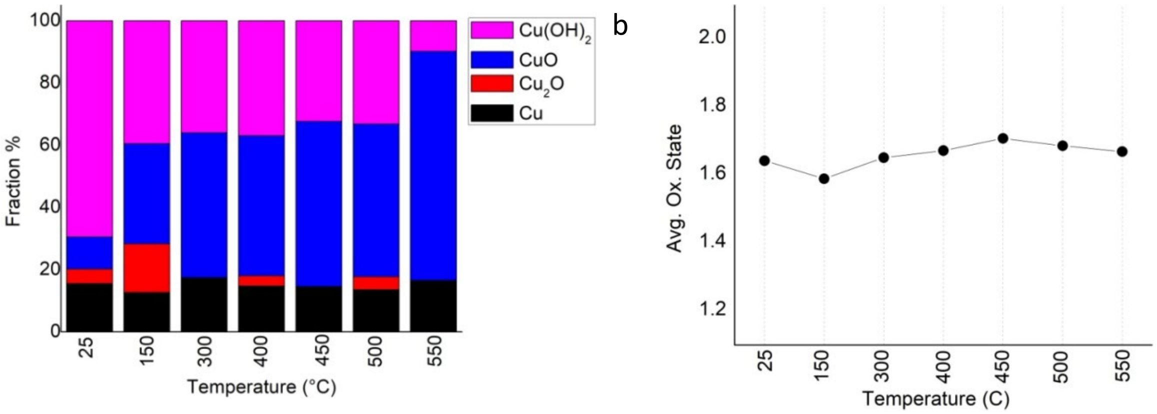

Figure S4. XANES analysis for Cu_4 clusters on UNCD supports at Cu K-edge (2%propane and 2% oxygen in He under 1.1 atm). (a) XANES spectra were collected at the Cu K-edge as shown in Fig. S3b and LCF fitting was performed with the Cu standards shown in Fig. S3 to obtain the composition.(b) Oxidation state of Cu in the Cu_4 clusters.

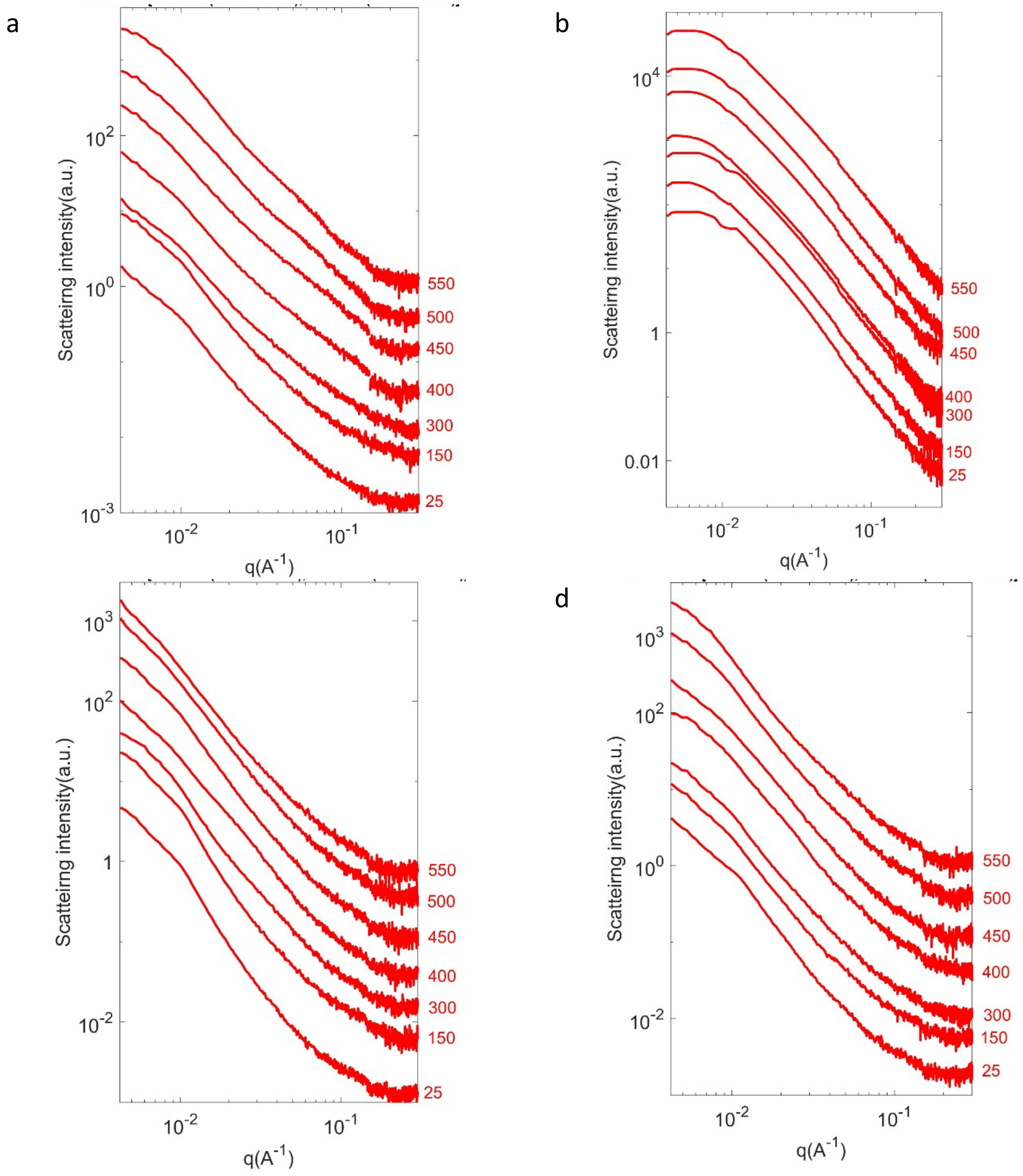

Figure S5. GISAXS spectra of supported Cu clusters during oxidative dehydrogenation of propane collected at 9.1 keV (2%propane and 2% oxygen in He under 1.1 atm). GISAXS spectra for (a) Cu_4 on alumina, (b) Cu_4 on UNCD, (c) Cu_{12} on alumina, (d) Cu_{20} on alumina.

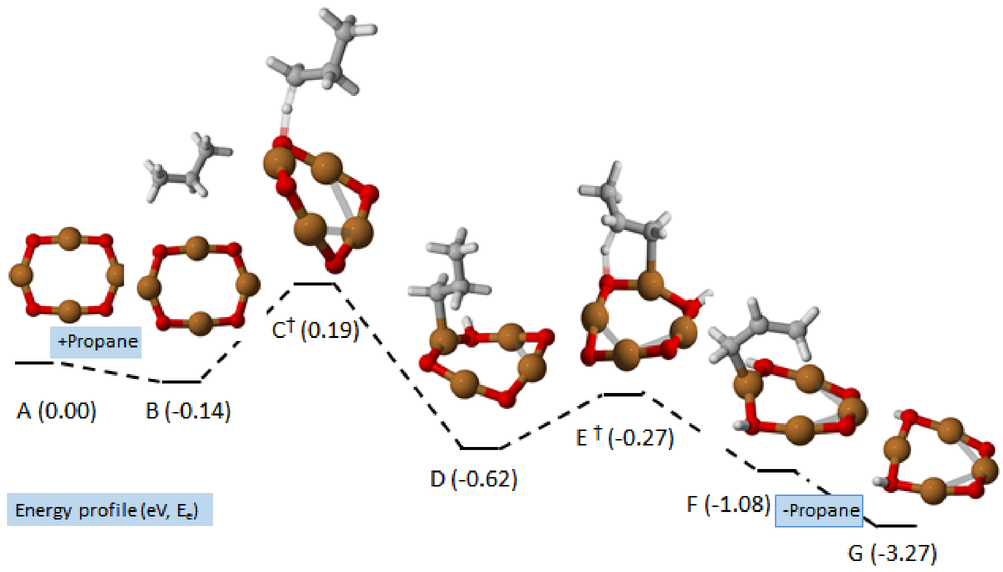

Figure S6. Computed total energy profile (not to scale) for the gas phase conversion of propane to propene by a Cu_4O_4 catalyst in the gas phase at the ωb97x-d/TZVPD level of theory. All energies are given in eV with reference to infinitely separated Cu_4O_4 and propane. The C† and E† are transition state structures. Note that the lowest energy state of Cu_4O_4 (A) corresponds to planar quintet state, while lowest energy structure of $Cu_4O_4H_2$ (G) corresponds to triplet electronic state.

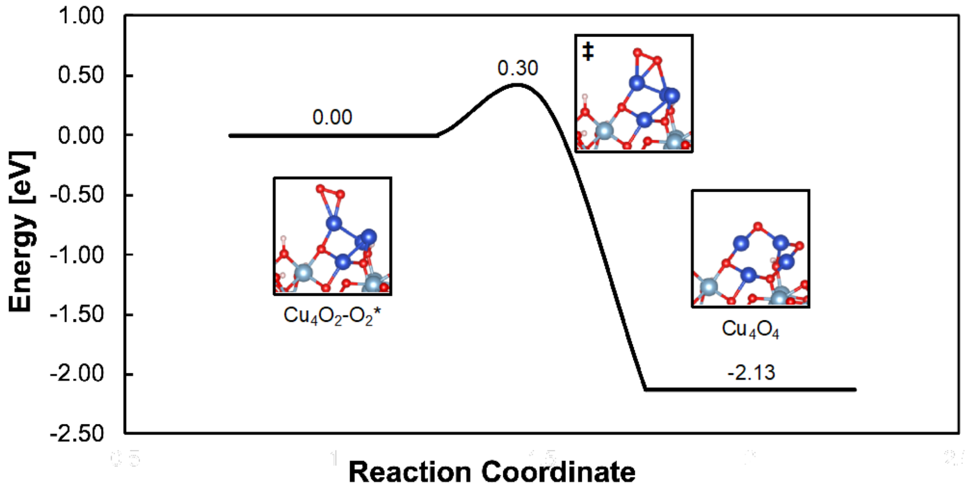

Figure S7. O_2 dissociation on hydroxylated amorphous alumina-supported Cu_4O_2, chosen as a representative model for the oxidized clusters, to recover the Cu_4O_4 cluster stoichiometry.

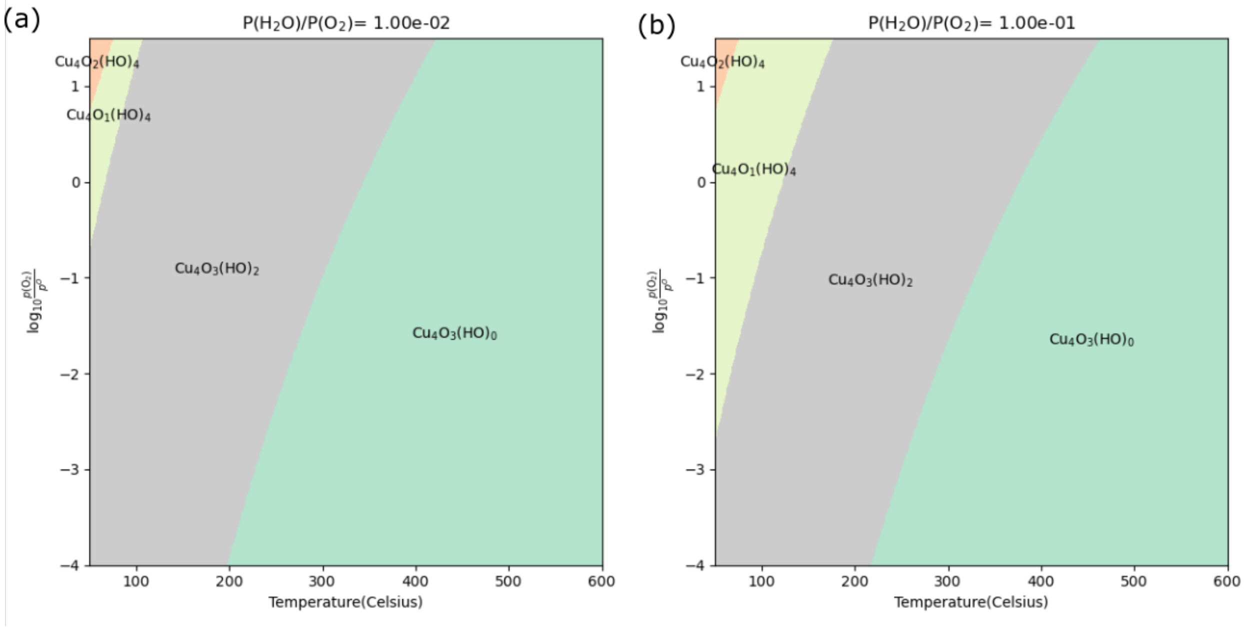

Figure S8. The phase diagram of the hydroxylated copper oxide cluster. The x-axis is the temperature in Celsius and y-axis is the partial pressure of oxygen in the log scale. The partial pressure of H_2O is assumed to be always 1 percent of the $P(O_2)$ (subfigure (a)) and 10 percent of $P(O_2)$ (subfigure (a)) respectively.

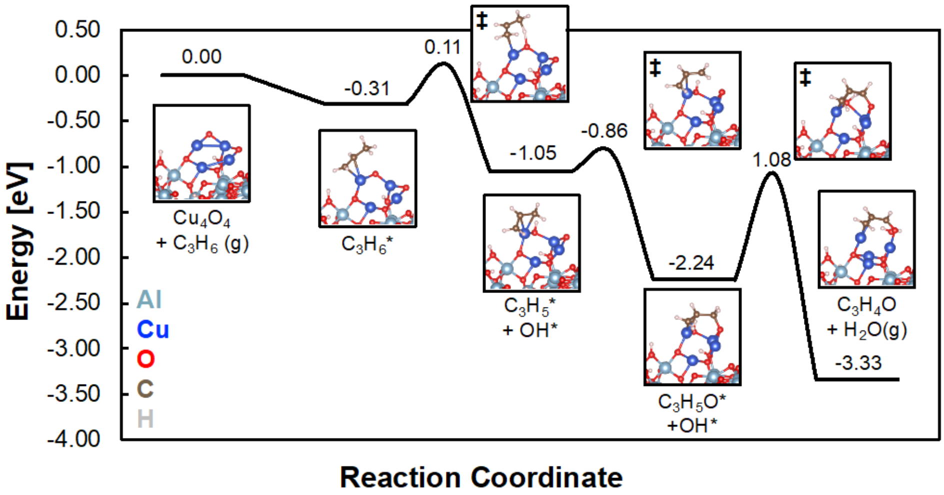

Figure S9. Energies of intermediates and transition states for the side reaction of acrolein formation from propylene.
